# Supplementary material for: Moderate white light exposure enhanced spatial memory retrieval by activating a central amygdala-involved circuit in mice
Source: Commun Biol. 2023 Apr 14;6:414. doi: 10.1038/s42003-023-04765-7 (PMC10104844; doi:10.1038/s42003-023-04765-7)
Supplement: Supplementary file 2 — Supplementary Information [file 42003_2023_4765_MOESM2_ESM.pdf]

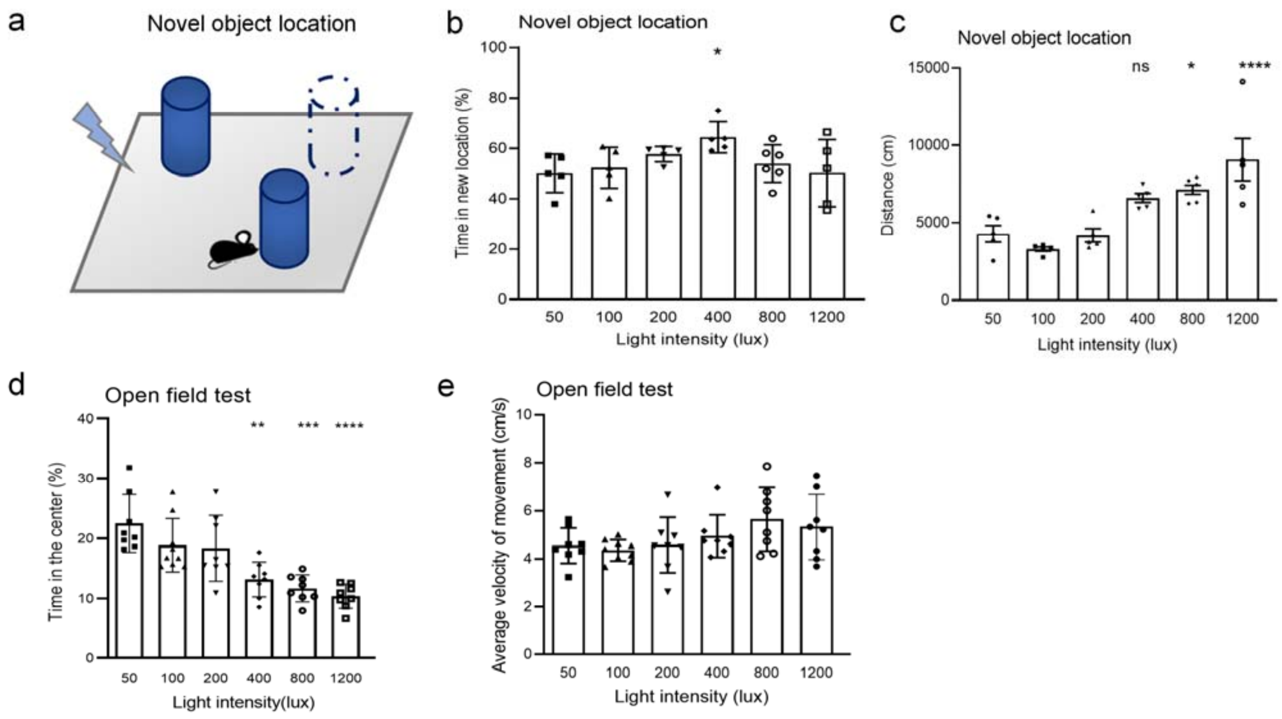

Supplementary Figure. 1 Effects of different intensities of white light exposure on behavioral performance in the novel object location (NOL) test and open field test (OFT). (a) The schematic diagram of the NOL test. (b) Percentage of time that mice spent at the object in the new location in the NOL test under different brightness of light exposure (n = 5-6, one-way ANOVA with Dunnett-t multiple comparison test). (c) Total distance traveled in the NOL test under different intensities of light exposure (n = 5-6, one-way ANOVA with Dunnett-t multiple comparison test). (d) Percentage of the time spent in the center area (n = 8-9, non-parametric Kruskal-Wallis H Test) in the OFT test under different brightness of light exposure. (e) Average movement speed (n = 8-9, non-parametric Kruskal-Wallis H Test) in the OFT test under different brightness of light exposure. In both tests, 50 lux light stimulation was used as a control. *P < 0.05; **P < 0.01; ***P < 0.001; ns, not significant. Data are expressed as mean ± SD.

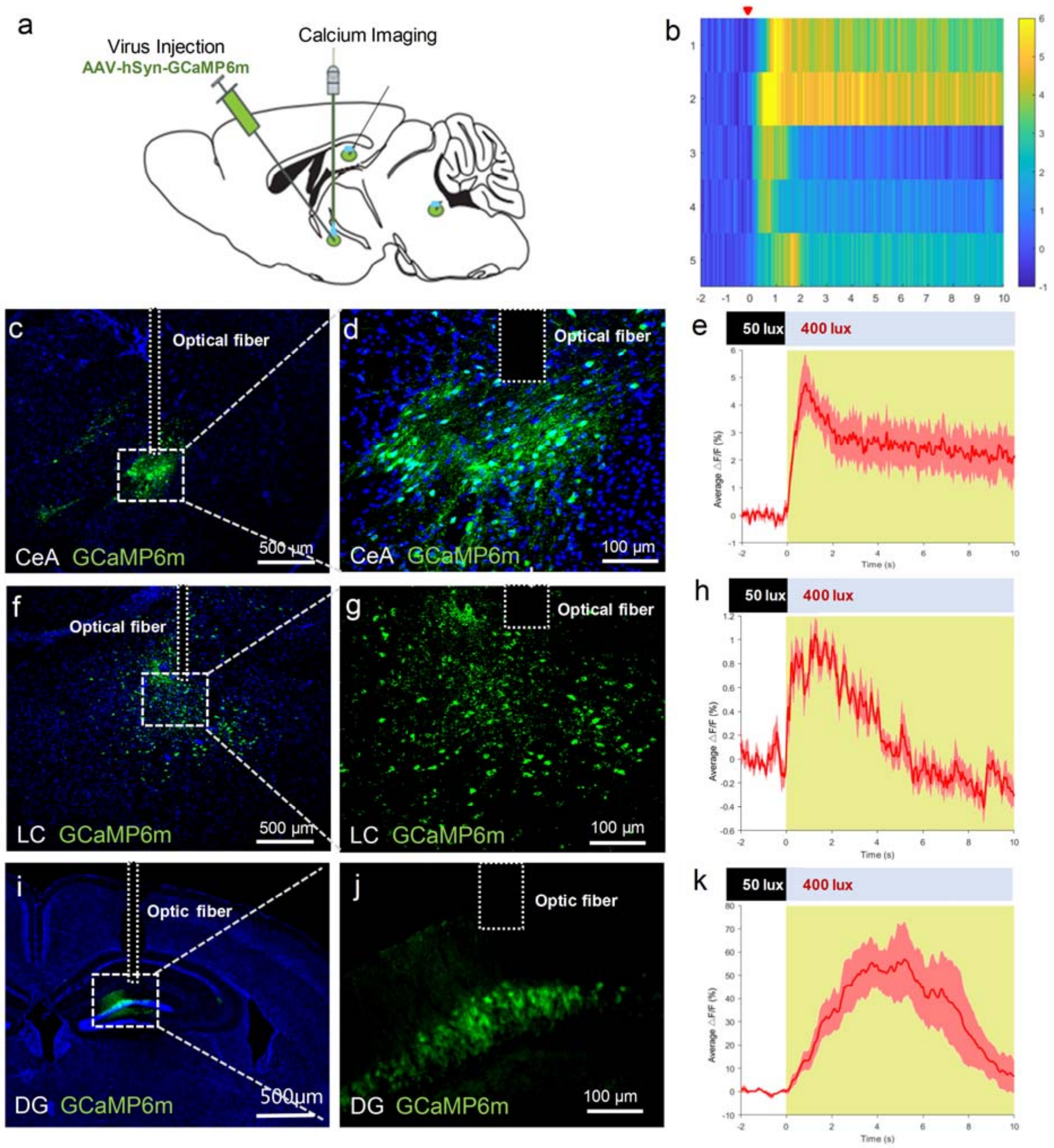

Supplementary Figure. 2 Neuronal activities were increased in the CeA, LC, and DG by 400 lux light exposure. (a) Schematic illustration of virus injections into the CeA, LC, or DG, and fiber optic implantation over the injection area for in vivo calcium imaging. (b) The heat map of calcium signal changes in the CeA after 400 lux light exposure in 5 mice. The red arrow indicates the start time of light exposure. (c-d) The expression of GCaMP6m in the CeA. The dotted lines represent the embedded optical fiber. The white-outlined area in c is shown at the higher magnification in d. (e) The averaged changes of calcium signal in the CeA after light exposure (n = 5). (f-g) The expression of GCaMP6m in the LC. The white-outlined area in f is shown at the higher magnification in g. (h) The averaged changes of calcium signal in the LC after light exposure (n = 5). (i-j) The expression of GCaMP6m in the DG. The white-outlined area in I is shown at the higher magnification in j. (k) The averaged changes of calcium signal in the DG after light exposure (n = 5).

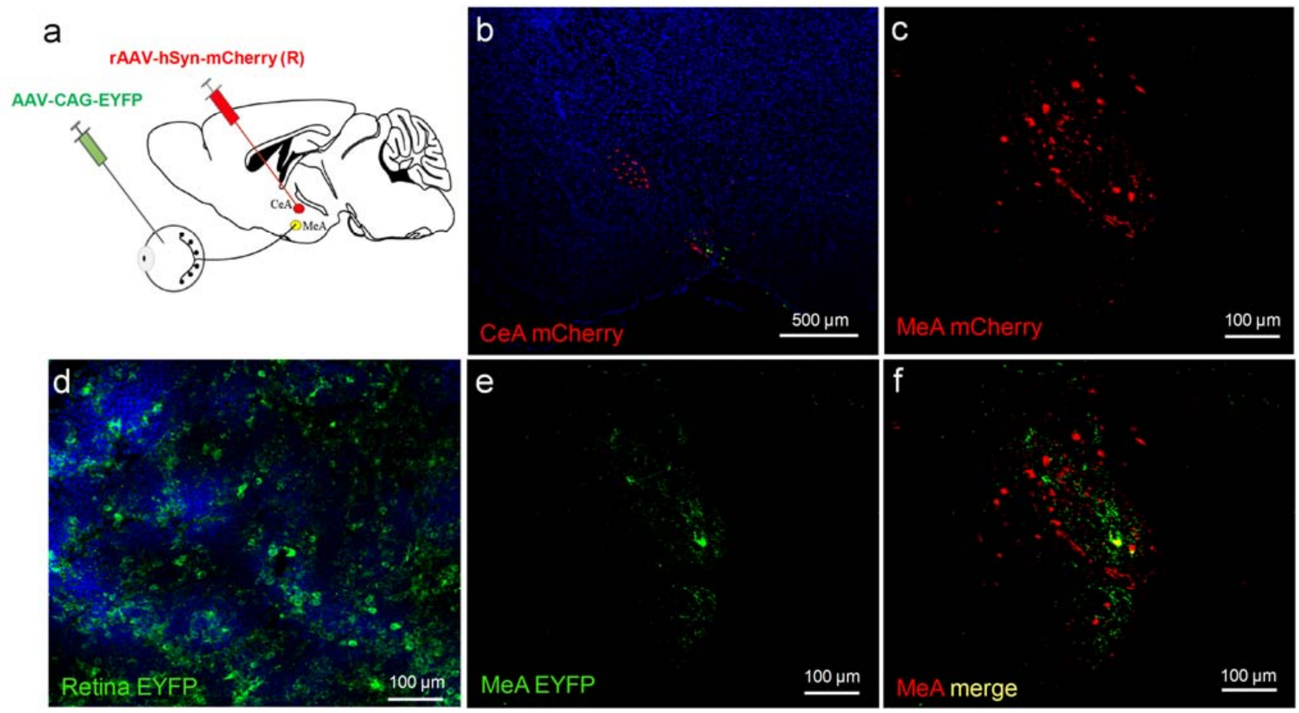

Supplementary Figure. 3 The amygdala (MeA) receives afferent inputs from the retina. (a) A schematic diagram of virus injection. The AAV-CAG-EYFP virus was injected into the eyeball, and the rAAV-hSyn-mCherry virus was injected into the CeA. (b) The expression of fluorescent mCherry proteins (red) in the CeA after intra-CeA injection of AAV-hSyn-mCherry virus. (c) The MeA neurons were retrogradely labeled by mCherry (red) after intra-CeA injection of the AAV-hSyn-mCherry virus. (d) The expression of EYFP (green) in the retina after injection of the AAV-CAG-EYFP virus into the eyeball. (e) The MeA neurons were anterogradely labeled with EYFT after the injection of the AAV-CAG-EYFP virus into the eyeball. (f) A composite (merged) image of c and e. White arrows depict cells that were co-labeled with mCherry and EYFP.

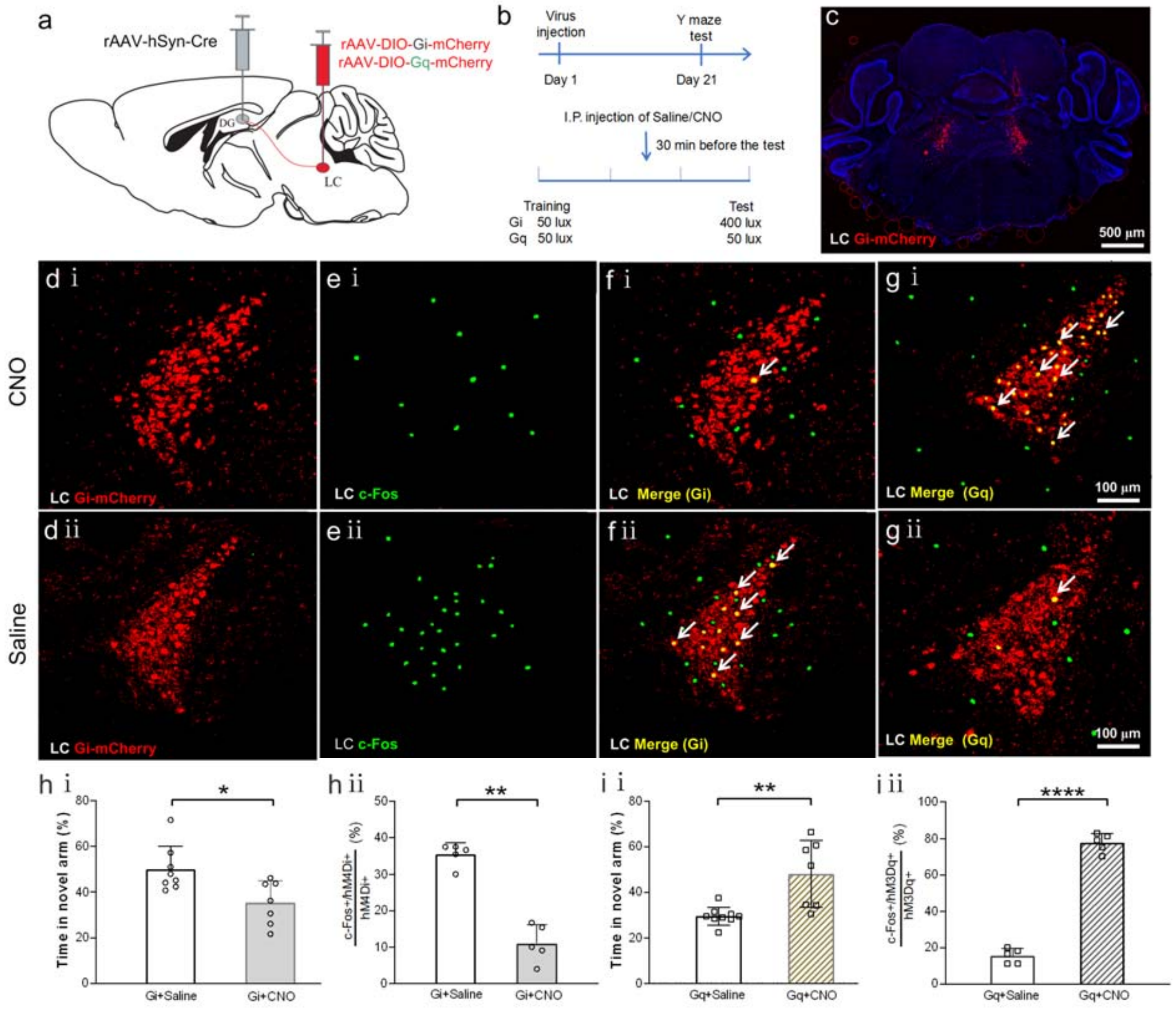

Supplementary Figure. 4 Activation of the LC-DG circuit plays a necessary and sufficient role in the enhanced spatial memory retrieval induced by 400 lux light exposure. a, Schematic illustration of injecting different chemogenetic viruses into the LC, and the injection of the rAAV-hSyn-Cre virus into the DG. b, Experimental protocol and timeline. c, The expression of mCherry in the LC after injection of the rAAV-DIO-Gi-mCherry virus into bilateral LC. d ⅰ and d ⅱ, mCherry is expressed in LC neurons in both CNO and saline groups. e ⅰ and e ⅱ, Representative images of immunofluorescence staining of c-Fos in LC neurons after intraperitoneal injection of CNO or saline. f ⅰ, The merged image of d ⅰ and e ⅰ. f ⅱ, The merged image of d ⅱ and e ⅱ. Cells co-labeled by mCherry and c-Fos were indicated by the white arrows. g ⅰ and g ⅱ, Representative images of c-Fos staining and expression of mCherry in the LC after intraperitoneal injection of CNO (g ⅰ) or saline (g ⅱ) in mice that received an intra-LC injection of the rAAV-DIO-Gq-mCherry virus, and that received an intra-DG injection of the rAAV-hSyn-Cre virus. Cells double-labeled by mCherry and c-Fos were indicated by white arrows. h ⅰ, In mice that express the inhibitory Gi-coupled receptors in the LC, CNO induced chemogenetic inhibition of CeA neurons and decreased the time spent exploring the novel arm, as compared to saline. The light treatment during the test was 400 lux. **h ⅱ,** CNO also reduced the number of c-Fos+ neurons in the LC (non-parametric Mann-Whitney U test). **i ⅰ,** In contrast, in mice that express the excitatory Gq-coupled receptors in the LC, CNO induced chemogenetic activation of LC neurons and increased the time spent exploring the novel

arm, as compared to saline (non-parametric Mann-Whitney U test). The light treatment during the test was 50 lux. **i** ii **,** CNO also increased the number of c-Fos+ neurons in the LC. *$P < 0.05$, **$P < 0.01$, ****$P < 0.0001$ vs. indicated group, two independent sample t-test unless otherwise noted, n = 7-9/group for behavioral test; n = 5 /group for c-Fos staining.

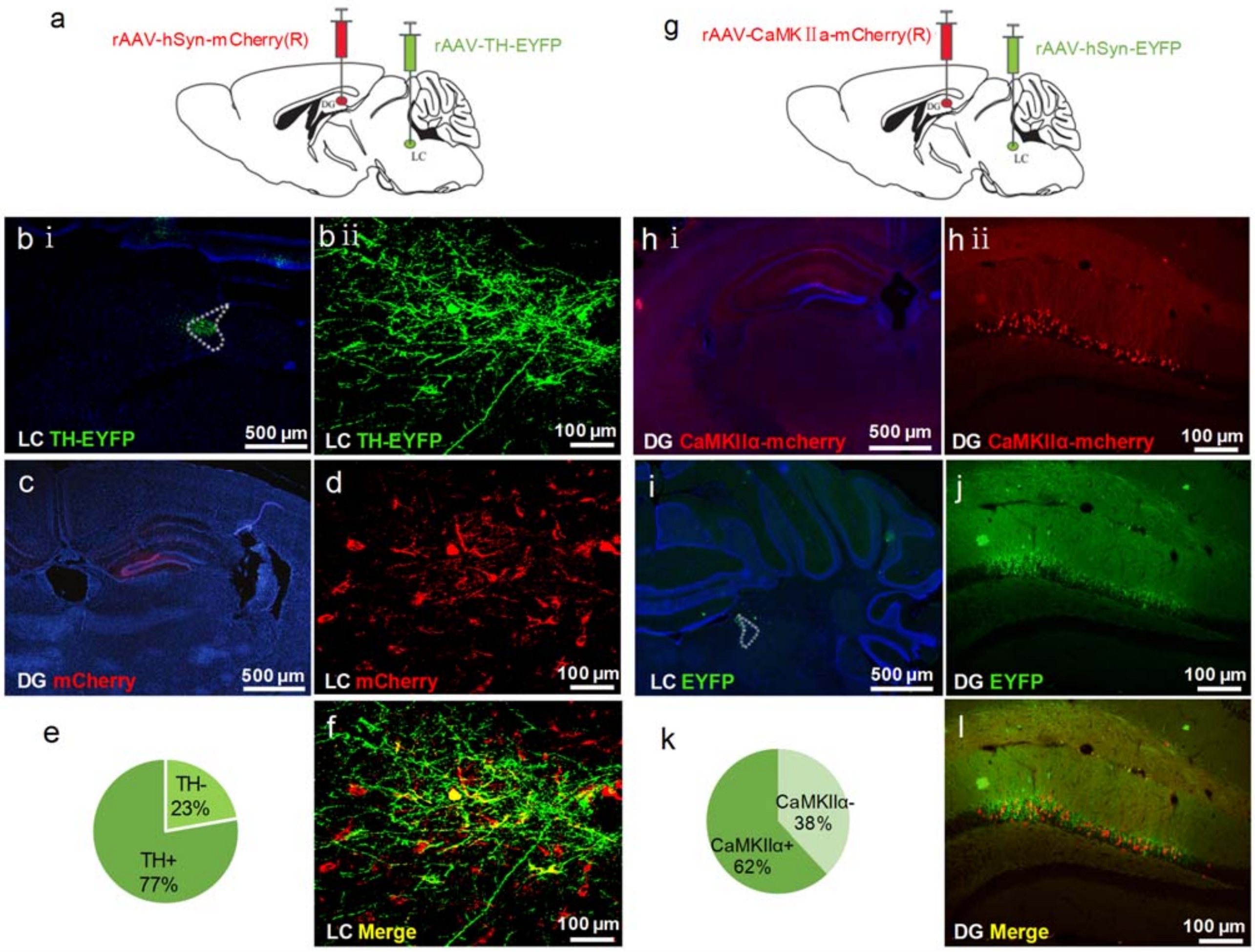

Supplementary Figure. 5 TH+ neurons in the LC predominantly project to CaMKII+ neurons in the DG. (a) The schematic diagram of intra-DG and intra-LC virus injections. (b i -b ii) The fluorescent EYFP proteins (green) showed a restricted expression in the LC after an intra-LC injection of the rAAV-TH-EYFP virus. (c) The fluorescent mCherry protein (red) was expressed in the DG after an intra-DG injection of the rAAV-hSyn-mCherry virus. (d) Neurons in the LC were retrogradely labeled with mCherry (red) after an intra-DG injection of the rAAV-hSyn-mCherry virus. (e) The proportion of TH+ (77%) and TH- (23%) LC neurons that project to the DG. (f) A composite (merged) image of b2 and d. Co-labeled neurons represent TH+ LC neurons that project to the DG. (g) A schematic diagram of virus injections. (h i -h ii) The fluorescent mCherry protein (red) was expressed in the DG after an intra-DG injection of the rAAV-CaMKIIα-mcherry virus. (i) The expression of fluorescent EYFP protein (green) in the LC after an intra-LC injection of the rAAV-hSyn-EYFP virus. (j) Neurons in the DG were anterogradely labeled with EYFP (green). (k) The proportion of CaMKIIα+ (62%), EYFP-labeled DG neurons that receive projections from the LC. (i) A merged image of h2 and j. Co-labeled neurons represent CaMKIIα+ DG neurons that receive projections from the LC.

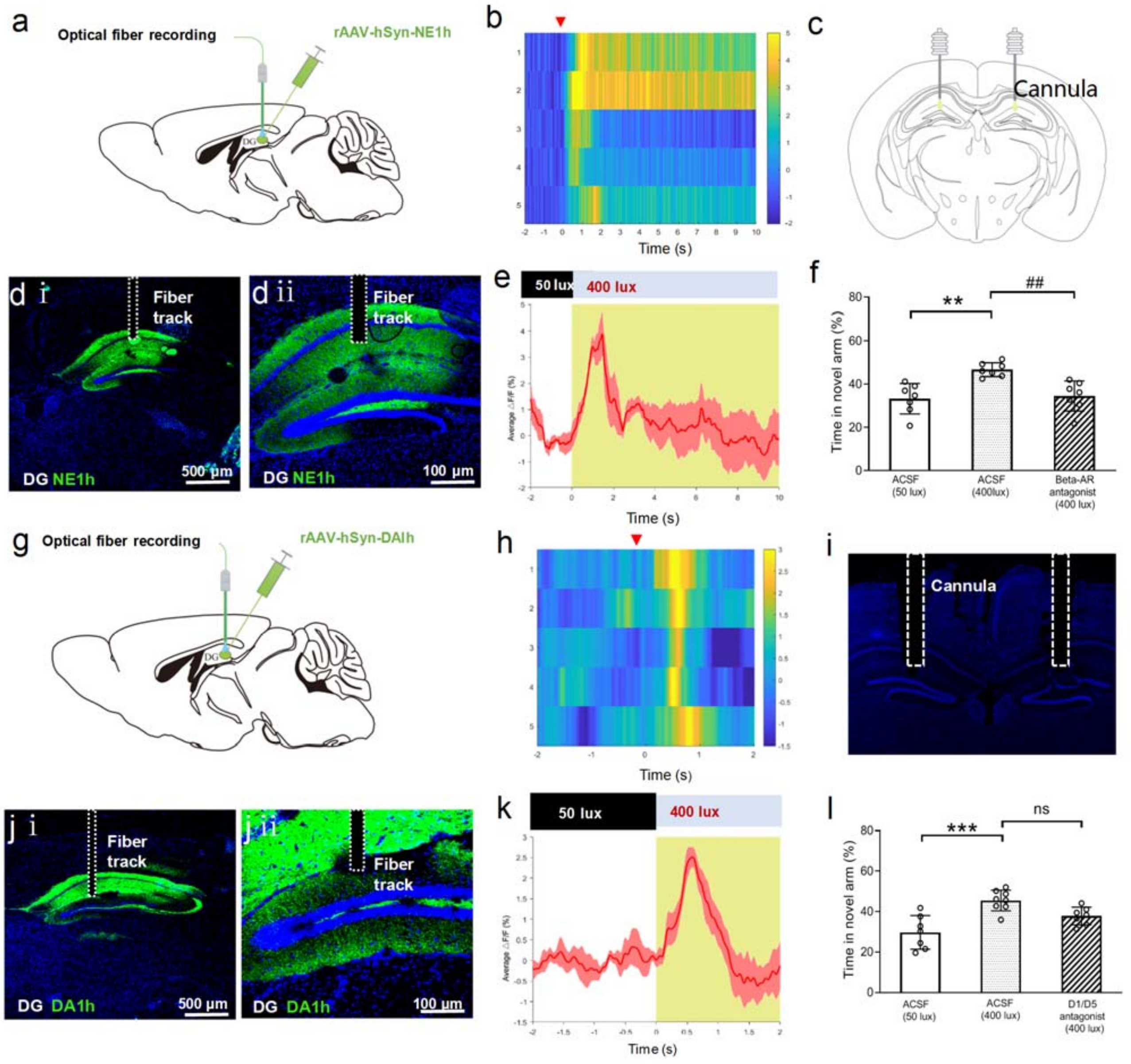

Supplementary Figure. 6 NE was released from TH+ LC-DG projection neurons and activated DG neurons to promote spatial memory retrieval. (a) The AAV-hSyn-NE1h virus was injected into the DG and an optical fiber was embedded above the injection site. (b) The heat map of NE signals in the DG after 400 lux light exposure in 5 mice. (c) The schematic diagram of cannulas embedded in bilateral DG. (dⅰ-dⅱ) The expression of NE1h induced by the AAV-hSyn-NE1h virus was restricted to the DG. The dotted lines represent the embedded position of the optical fiber. (e) The averaged NE signals that were induced by light exposure (n = 5). (f) Quantification of behavioral data in the Y maze test. (g) The AAV-hSyn-DA1h virus was injected into the DG. (h) The heat map of changes of DA signal after 400 lux light exposure in 5 mice. (i) The dotted lines in the coronal brain sections show the cannulas embedded in bilateral DG. (jⅰ-jⅱ) The restricted expression of DA1h in the DG. The dotted lines represent the position of the embedded optical fiber. (k) The averaged DA signal in response to 400 lux light exposure (n = 5). (l) Quantification of time spent in the novel arm in the Y maze test. (f and l): ACSF (50 lux): 50 lux light exposure was adopted during the training and testing periods, and ACSF was infused into the DG 10 min before the testing period; ACSF (400 lux):

50 lux light exposure was adopted during the training period, and 400 lux light was used during the testing period, and ACSF was infused into the DG 10 min before testing. Beta-AR antagonist or D1/D5 antagonist (400 lux): 50 lux light exposure was adopted during the training period and 400 lux light was applied during the testing period. The antagonist was infused into the DG 10 min before testing. Data are expressed as mean ± SD. ns: not significant, $^{**}P < 0.01$, $^{\#\#}P < 0.001$, $^{***}P < 0.0001$ vs. indicated group, one-way ANOVA Dunnett-t multiple comparisons test, n = 7/group.
