# Supplementary material for: Moderate white light exposure enhanced spatial memory retrieval by activating a central amygdala-involved circuit in mice
Source: Commun Biol. 2023 Apr 14;6:414. doi: 10.1038/s42003-023-04765-7 (PMC10104844; doi:10.1038/s42003-023-04765-7)
Supplement: Supplementary file 3 — Description of Additional Supplementary Files [file 42003_2023_4765_MOESM3_ESM.pdf]

Description of Additional Supplementary Files

File name: Supplementary Data
Description: The source data underlying Figs. 1a2, 1b2, 1c-d, 2i, 4h1-i2, 6f, Supplementary Figs. S1b-e, S4h1-i2, S6f, l.
